# In Vitro Selection of *Lactobacillus* and *Bifidobacterium* Probiotic Strains for the Management of Oral Pathobiont Infections Associated to Systemic Diseases

**DOI:** 10.3390/ijms232416163

**Published:** 2022-12-18

**Authors:** Paola Zanetta, Diletta Francesca Squarzanti, Alessia di Coste, Roberta Rolla, Paolo Aluffi Valletti, Massimiliano Garzaro, Valeria Dell’Era, Angela Amoruso, Marco Pane, Barbara Azzimonti

**Affiliations:** 1 Laboratory of Applied Microbiology, Department of Health Sciences (DiSS), Center for Translational Research on Allergic and Autoimmune Diseases (CAAD), School of Medicine, Università del Piemonte Orientale (UPO), Corso Trieste 15/A, 28100 Novara, Italy; 2 Clinical Chemistry Unit, DiSS, School of Medicine, University Hospital “Maggiore della Carità”, Università del Piemonte Orientale (UPO), Corso Mazzini 18, 28100 Novara, Italy; 3 ENT Division, DiSS, School of Medicine, University Hospital “Maggiore della Carità”, Università del Piemonte Orientale (UPO), Corso Mazzini 18, 28100 Novara, Italy; 4 Probiotical Research Srl, Via Mattei 3, 28100 Novara, Italy

**Keywords:** *Aggregatibacter actinomycetemcomitans*, *Streptococcus* spp., *Lactobacillus* spp., *Bifidobacterium* spp., probiotics, probiotic cell-free supernatants, oral infection-associated diseases

## Abstract

The human oral pathobionts *Aggregatibacter actinomycetemcomitans*, *Streptococcus mitis* and *Streptococcus mutans*, in dysbiosis-promoting conditions, lead to oral infections, which also represent a threat to human systemic health. This scenario may be worsened by antibiotic misuse, which favours multi-drug resistance, making the research on pathogen containment strategies more than crucial. Therefore, we aimed to in vitro select the most promising probiotic strains against oral pathogen growth, viability, biofilm formation, and co-aggregation capacity, employing both the viable probiotics and their cell-free supernatants (CFSs). Interestingly, we also assessed probiotic efficacy against the three-pathogen co-culture, mimicking an environment similar to that in vivo. Overall, the results showed that *Lactobacillus* CFSs performed better than the *Bifidobacterium*, highlighting *Limosilactobacillus reuteri* LRE11, *Lacticaseibacillus rhamnosus* LR04, *Lacticaseibacillus casei* LC04, and *Limosilactobacillus fermentum* LF26 as the most effective strains, opening the chance to deeper investigation of their action and CFS composition. Altogether, the methodologies presented in this study can be used for probiotic efficacy screenings, in order to better focus the research on a viable probiotic, or on its postbiotics, suitable in case of infections.

## 1. Introduction

In the last years, oral microbiota have been gaining more and more interest, not only for their importance in the maintenance of oral health but also because they have been found crucial for the systemic one [1,2]. While it is well established that their unbalanced composition leads to local infective diseases, such as caries, gingivitis, and periodontitis [3], also promoting oral cancer onset and progression [4], research is now focusing more on the oral microbiota interplay on general human health [5,6], since oral micro-organisms can spread through the human body.

The investigation was focalized on the three oral commensals *Aggregatibacter actinomycetemcomitans*, *Streptococcus mitis* and *Streptococcus mutans* that, in dysbiosis-promoting conditions, can become opportunistic pathogens and behave as pathobionts. 

*A. actinomycetemcomitans* is a low-abundant Gram-negative pathobiont of the oral cavity that can cause chronic local and systemic inflammatory disorders, such as periodontitis, tooth loss, atherosclerosis, and oropharyngeal pre- and tumoral lesions [7]. It is a member of the HACEK group (*Haemophilus* spp., *A. actinomycetemcomitans*, *Cardiobacterium hominis*, *Eikenella corrodens*, and *Kingella kingae*) which is responsible for at least the 1.3% of all infective endocarditis (IE) cases [8]. The commensal *S. mitis*, through adhesins, proteases, and toxins, can cause severe human infections, such as the streptococcal toxic shock syndrome in a healthy adult, as recently described in a case report [9]. Moreover, its ability to easily translocate across the epithelial barrier allows it to enter into the bloodstream, promoting severe complications, such as septicaemia, bacteraemia, and IE [10,11]. The cariogenic *S. mutans* can reside on tooth surfaces and, when incorrect eating habits and poor oral hygiene allow its overgrowth, caries and extraoral pathologies, such as cerebral microbleeds, atherosclerosis, and IE, develop [12,13,14]. Bacteria of the *Streptococcus* genus have been observed to frequently cause bloodstream infections responsible for IE recurrence. Interestingly, *S. mitis* and *S. mutans* are two of the main species causing the highest IE prevalence [15].

Prophylactic and therapeutic antibiotic misuse in the oral praxis is highly contributing to oral microbiota imbalance and the global emergence of multi-drug resistance (MDR), promoting the overgrowth, persistence, and virulence of pathobiont bacteria and increasing the chances for the human host to develop extra-oral pathologies [10,11].

A growing body of literature is highlighting probiotic potential as tools to better control pathobiont overgrowth, virulence, and infectivity, also thanks to their interaction with the other microbial members [16,17], preventing and slowing down the onset of pathogenic bacteria MDR and infection-related disorders. However, their application in the prevention and treatment of caries, periodontitis, gingivitis, and related systemic diseases, is still under investigation. 

Six viable lactic acid bacteria (LAB; *Levilactobacillus brevis* LBR01, *Ligilactobacillus salivarius* LS03, *Limosilactobacillus reuteri* LRE11, *Lacticaseibacillus rhamnosus* LR04, *Lacticaseibacillus casei* LC04, *Limosilactobacillus fermentum* LF26) and two *Bifidobacterium* strains (*Bifidobacterium longum* 04 and *B. breve* B632), and their cell-free supernatants (CFSs) were used, with the aim to select the ones that, in in vitro experiments, can better contain *A. actinomycetemcomitans*, *S. mitis*, and *S. mutans* growth, viability, biofilm formation, and co-aggregation ability. Interestingly, all the probiotics displayed different containment activities when employed as viable strains or with their CFSs. Specifically, in the agar spot test, viable *L. rhamnosus* LR04 and *B. longum* 04 showed a complete growth inhibition activity against *A. actinomycetemcomitans* and *S. mutans*, while they determined the higher inhibition halos against *S. mitis*. On the other hand, *L. reuteri* LRE11 CFS demonstrated the best activity in reducing pathogen viability. Biofilm formation was prevented by all CFSs, except by the ones of *L. brevis* LBR01, which were never effective, and *L. salivarius* LS03, which was not effective against *A. actinomycetemcomitans* and *S. mitis*. 

To better define viable probiotic and CFS activity in a complex bacterial environment, the same experiments were performed combining all three pathogens, also developing an auto- and co-aggregation assay, to determine whether live probiotics and their CFSs could interfere with pathogen interactions. In general, although the containment effect of viable probiotic strains was milder, *L. rhamnosus* LR04 and *L. casei* LC04 completely inhibited the three-pathogen co-culture growth, while *L. reuteri* LRE11 CFS was still the best in reducing the three-pathogen viability. All the CFSs could prevent the pathogen co-culture biofilm formation, except for *L. brevis* LBR01 and *L. salivarius* LS03 CFSs, which were ineffective, and *B. breve* B632 CFS, which slightly lost its effectiveness over time. The co-aggregation assay revealed that viable probiotics seemed to interact with the three pathogens, in some cases increasing the co-aggregation percentage; while only the CFSs of *L. brevis* LBR01 and *L. salivarius* LS03 significantly reduced the co-aggregation levels of the three pathogens, with LBR01 CFS displaying a specific inhibition. 

This work paves the way for probiotic employment in oral infection control, preventing systemic disease onset, such as severe IE, still associated with high mortality rates [18], and limiting antibiotic misuse to prevent further MDR occurrence.

## 2. Results and Discussion

### 2.1. Agar Spot Test

**Figure 1:**
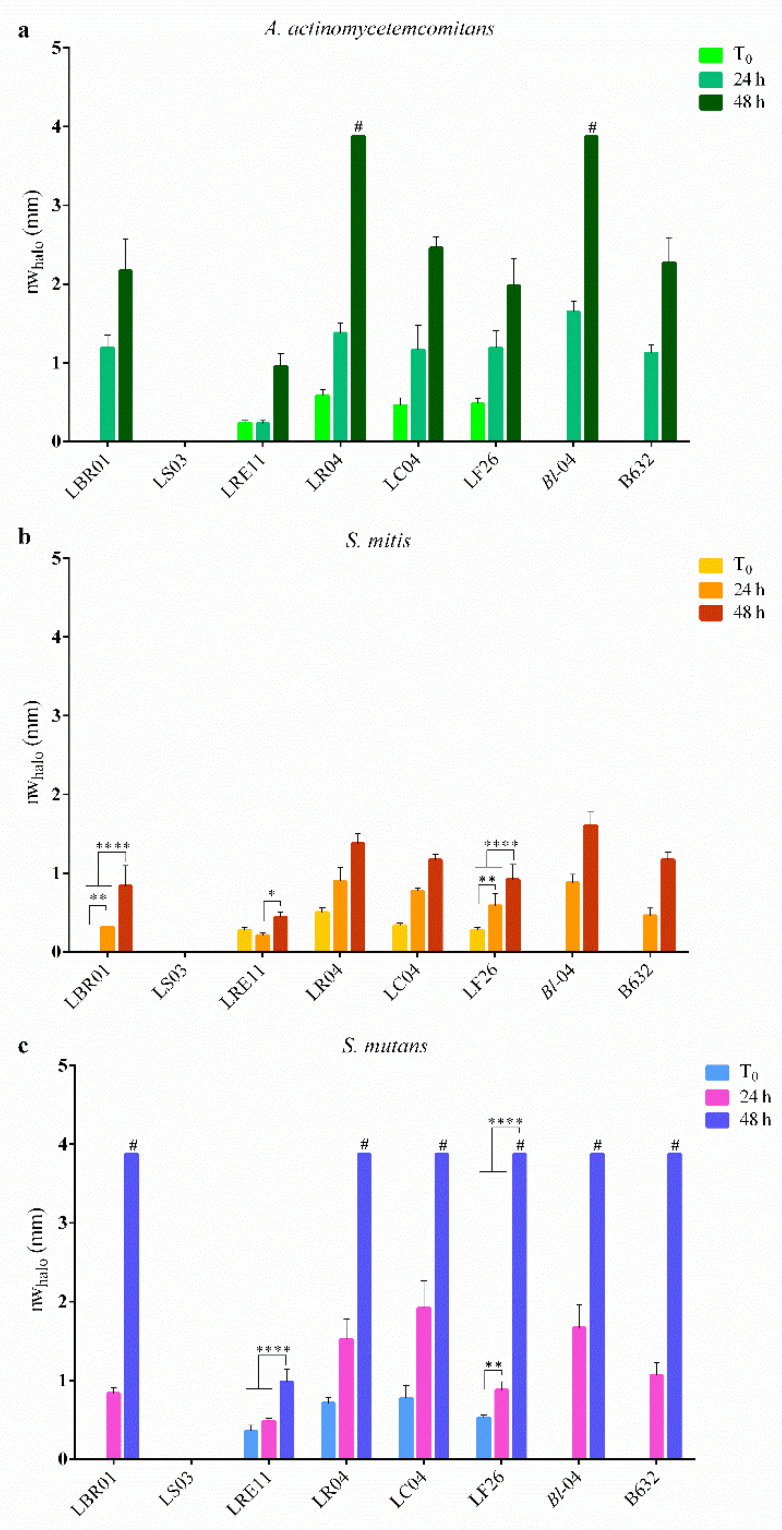
Normalized width measurements of inhibition halos obtained in the agar spot test. The diameters in mm of the inhibition halos were measured after 48 h of pathogen incubation with the probiotic spots without (T_0_), or with 24 and 48 h pre-incubation. In the graphs, the normalized width halo (nw_halo_) is represented for (**a**) *A. actinomycetemcomitans*, (**b**) *S. mitis*, and (**c**) *S. mutans*. Data are expressed as the mean of three independent experiments ± SD. # complete growth inhibition zones. * *p* < 0.05; ** *p* < 0.01; **** *p* < 0.0001.

The agar spot test was used to determine viable probiotic effects in containing oral pathogen growth. The normalized width halo (nw_halo_) measurements (mm) ± SD are listed and represented in Table 1 and Figure 1. LBR01, *B. longum* 04, and B632 were ineffective against the three pathogens only at T_0_, while LS03 was never effective in inhibiting their growth, independently from the spot incubation time before pouring the pathogen suspension (Figure 1a–c). For all the other probiotics, the inhibition halo diameter increased over time. Specifically, all the probiotics, except LS03 at all incubation times, and LRE11 at T_0_ versus 24 h, showed a significant increase in growth inhibition against *A. actinomycetemcomitans*, according to the spot incubation time (Figure 1a; *p* < 0.0001). LR04 and *B. longum* 04 completely inhibited *A. actinomycetemcomitans* growth only when pre-incubated for 48 h before the pathogen plating (Figure 1a). On the other hand, none of the probiotics completely inhibited the growth of *S. mitis* (Figure 1b). An increased growth inhibition over time was also observed against this pathogen but not as wide as the one noticed in *A. actinomycetemcomitans*. Except for LRE11 and LF26, whose significant differences are shown in Figure 1b, all the other probiotics showed a significant increase in the inhibition zone diameter accordingly to the spot incubation time (*p* < 0.0001). Figure 1c shows live probiotic effects against *S. mutans* growth. The trend was the same observed for the other two pathogens, with the difference that LBR01, LR04, LC04, LF26, *B. longum* 04, and B632 displayed a complete pathogen growth inhibition when incubated for 48 h before performing the assay. Huge significant differences were observed in the increase of the inhibition zones over time (*p* < 0.0001), with some exceptions shown in Figure 1c.

*L. rhamnosus* and *L. casei* efficacy towards the growth of *A. actinomycetemcomitans* and *S. mutans* was also observed by Gönczi and colleagues in an agar diffusion assay, where they found that *L. rhamnosus* was most effective against *S. mutans*, while *L. casei* was effective against *A. actinomycetemcomitans* [19]. Coman and colleagues showed *L. rhamnosus* IMC501^®^ efficacy against *S. mutans* in four in vitro experimental models, underlying how pathogen inhibition depends not only on pathogen sensitivity towards the antimicrobials produced by the specific probiotic but also on the method used [20]. Another work also highlighted the capacity of *L. fermentum* TcUESC01 to inhibit *S. mutans* growth only between 14 and 16 h [21], while our LF26 showed a complete growth inhibition of this pathogen when the probiotic was pre-incubated for 48 h before *S. mutans* plating. Teanpaisan and collaborators demonstrated that *L. rhamnosus* SD5, *L. casei* SD2, and *L. salivarius* SD3, inhibited the growth of both *S. mutans* and *A. actinomycetemcomitans* in an agar overlay method [22]. Finally, Jang and colleagues found that *L. brevis* KU15153 spots pre-incubated for 24 h were the best in containing *S. mutans* KCTC 5316 growth in an agar spot assay [23], while our LBR01 completely inhibited this pathogen when pre-incubated for 48 h. To our knowledge, no agar spot data are available in the literature against *S. mitis*.

### 2.2. Viability Assay

In order to determine if postbiotics can reduce oral pathogen viability, probiotic CFSs were produced, and a BacTiter-Glo^TM^ Microbial Cell Viability Assay was performed. 

**Figure 2:**
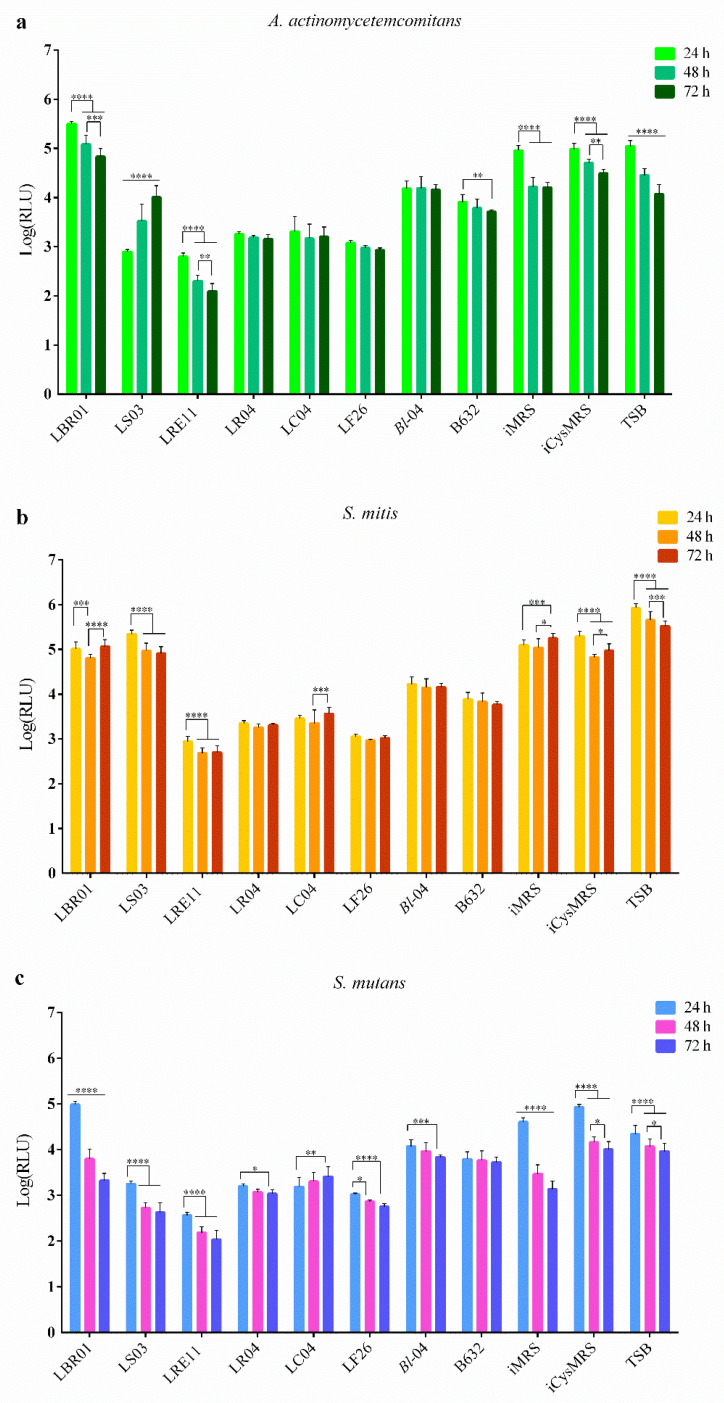
Viability assay. (**a**) *A. actinomycetemcomitans*, (**b**) *S. mitis*, and (**c**) *S. mutans* viability was determined after 24, 48, and 72 h of probiotic CFS treatment. Data are represented as the Log(mean) of three independent experiments ± SD. * *p* < 0.05; ** *p* < 0.01; *** *p* < 0.001; **** *p* < 0.0001. Log(RLU) = Logarithm10 (relative luminescence unit); CFS = cell-free supernatant.

LRE11 CFS showed the greatest inhibitory activity against *A. actinomycetemcomitans* compared to the other LAB and the *Bifidobacterium* strains, with an increased effect over time, as shown by the statistics reported in the graph (Figure 2a). LBR01, LS03, *B. longum* 04, and B632 CFSs were not as effective as LR04, LC04, and LF26 ones. LBR01 CFS was the most ineffective and, even though *A. actinomycetemcomitans* viability significantly decreased over time, it was always higher when compared to the three controls at all the times tested (*p* < 0.0001, Figure 2a). LS03 CFS, instead, reduced the pathogen viability only at 24 h similarly to LRE11 CFS at the same time point; then the effect was significantly lost over time, maintaining a statistically significant reduction at 72 h only in comparison to the incubated MRS with cysteine (iCysMRS) control (*p* < 0.0001, Figure 2a). Among the two *Bifidobacterium* CFSs, B632 was better in reducing *A. actinomycetemcomitans* viability when compared to the one of *B. longum* 04 (*p* < 0.01 at 24 h, and *p* < 0.0001 at 48 and 72 h, Figure 2a). Among the control conditions, at 24 h no significant differences were observed, with a common decrease in the pathogen viability over time. Specifically, a stronger reduction was observed in incubated MRS (iMRS), with a significant difference at 48 h versus tryptic soy broth (TSB; *p* < 0.001, Figure 2a) and iCysMRS (*p* < 0.0001, Figure 2a). At 48 h the difference between TSB and iCysMRS was also significant (*p* < 0.001, Figure 2a). At 72 h, instead, no significant difference was observed between iMRS and TSB, while it was present when comparing iMRS with iCysMRS (*p* < 0.001, Figure 2a), and TSB with iCysMRS (*p* < 0.0001, Figure 2a). 

Similar activity patterns were detected for *S. mitis* (Figure 2b). LRE11 CFS showed the best activity in reducing this pathogen viability when compared to all the other CFSs and the controls at all the evaluated time points (*p* < 0.0001, Figure 2b), with the only exception at 24 h, when no difference was observed between LRE11 and LF26 CFSs. LBR01 and LS03 CFSs were the least effective treatments and showed a similar activity with the only difference observed between them at 24 h, where LS03 showed a significantly higher viability than LBR01 (*p* < 0.001, Figure 2b). Again, *B. longum* 04 and B632 CFSs were not as effective as LAB CFSs (*p* < 0.0001 at all endpoints, Figure 2b), with B632 CFS being the most effective when compared to the one of *B. longum* 04 (*p* < 0.0001 at all endpoints, Figure 2b). At all the tested times, controls displayed a significantly higher pathogen viability when compared to CFS treatments (*p* < 0.0001, Figure 2b), except for the ones of LBR01 and LS03, and the MRS controls. MRS controls resulted in a significant decrease over time of *S. mitis* viability with respect to TSB at all measuring points (*p* < 0.0001, Figure 2b). 

LRE11 CFS was the most effective treatment also against *S. mutans* (*p* < 0.0001 at all the time points and versus all conditions, including controls, Figure 2c), displaying an increased viability reduction over time. In this case, also LS03 CFS revealed its effectiveness in reducing pathogen viability over time, with a similar activity to LF26 CFS (no statistically significant differences were observed between these two conditions at all the endpoints). LBR01 CFS at 24 h was still the less effective against *S. mutans* with a significant difference when compared to other treatments and the controls (*p* < 0.0001, Figure 2c), except for iCysMRS (not significant: ns). Between *B. longum* 04 and B632 CFSs, no significant differences were observed, except at 24 h (*p* < 0.0001, Figure 2c). *S. mutans* showed a viability reduction over time in the controls, which, at all the time points, displayed significant differences (*p* < 0.0001, Figure 2c) among them, except for iCysMRS versus TSB at 48 and 72 h (ns).

To summarize, LBR01 CFS was the one showing the lowest activity against the single pathogens, followed by LS03 CFS, which significantly reduced only *S. mutans* viability over time, while its effect observed against *A. actinomycetemcomitans* at 24 h was lost at the other endpoints. Nissen and colleagues also observed that *L. salivarius* OMZ520 CFS did not affect *A. actinomycetemcomitans* growth, but they found that it strongly attenuated the pathogen expression of leukotoxin and cytolethal distending toxin in a time-dependent manner [24], while *L. rhamnosus* Lr32 CFS showed the same effect, together with a partial biofilm biomass inhibition [25]. Differently from what was observed with viable LRE11, its CFS was the best when compared to all the others in reducing pathogen viability, significantly increasing its efficacy over time. In a recent work, live *L. reuteri* ATCC PTA 5289 and DSM17938 were revealed to be the best probiotic strains to contain oral pathogens growth, including *A. actinomycetemcomitans* [26]. The CFSs of LR04, LC04, and LF26 showed similar and stable effects, with only that of LF26 improving over time versus *S. mutans*, and, in general, it was better than the one showed by the *Bifidobacterium* CFSs. Among the two Bifidobacteria, B632 CFS was slightly better than the one of *B. longum* 04 in reducing the single pathogen viabilities. In this regard, Lee and collaborators showed that *S. mutans* and *A. actinomycetemcomitans* viability could be affected by viable *Bifidobacterium* spp.; in particular, *B. adolescentis* SPM1005 strongly reduced *S. mutans* viability [27].

### 2.3. Biofilm Formation Assay

**Figure 3:**
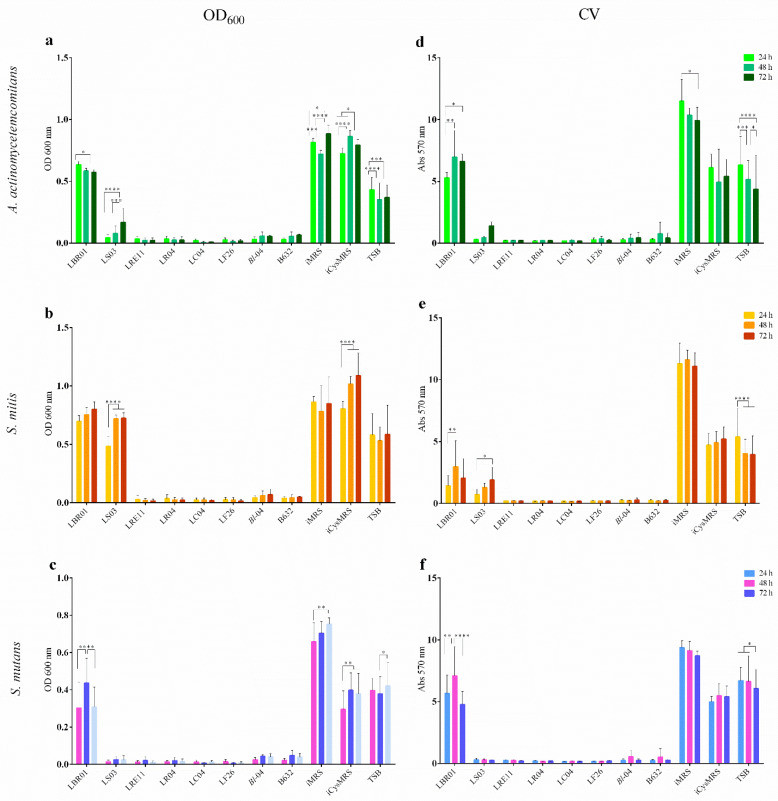
Optical density (OD) measurement at 600 nm and crystal violet (CV) biofilm quantification. CFSs were assayed to determine their ability in preventing pathogen biofilm formation through OD_600_ measurements for (**a**) *A. actinomycetemcomitans*, (**b**) *S. mitis*, and (**c**) *S. mutans*; and CV quantification for (**d**) *A. actinomycetemcomitans*, (**e**) *S. mitis*, and (**f**) *S. mutans*. Data are represented as the mean of three independent experiments ± SD. * *p* < 0.05; ** *p* < 0.01; *** *p* < 0.001; **** *p* < 0.0001. OD 600 nm = optical density at 600 nm; Abs 570 nm = absorbance at 570 nm; CFSs = cell-free supernatants.

**Figure 4:**
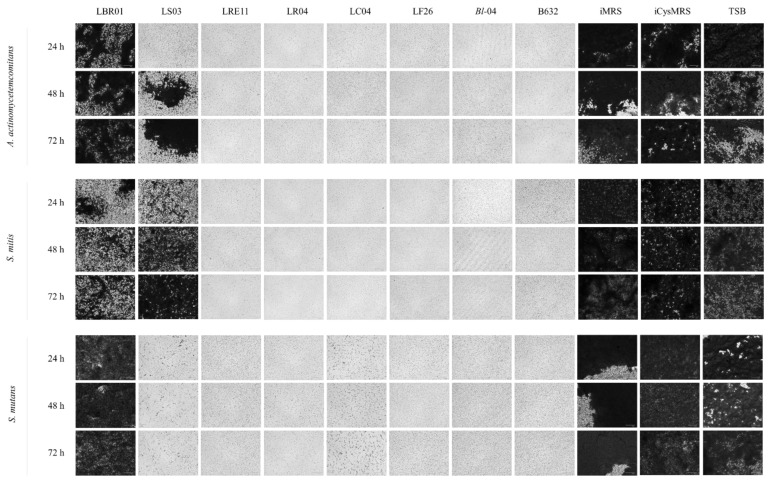
Representative crystal violet-stained biofilm images. Images were obtained at FLoid^TM^ Cell Imaging Station. Magnification 460×.

This assay was conducted to assess the probiotic CFS ability to reduce pathogen biofilm formation. Optical density at 600 nm (OD_600_) measurements revealed that all pathogen biofilms grew well in each control, with LBR01 CFS being completely ineffective against all of them (*p* < 0.0001 versus all treatments at all endpoints; Figure 3a, b, and c). LS03 CFS was ineffective against *S. mitis* with significant differences when compared to the other CFSs (*p* < 0.0001; Figure 3b), while it showed a high efficacy against *S. mutans* (Figure 3c), with no significant differences versus all the other conditions. On the other hand, against *A. actinomycetemcomitans*, LS03 CFS effect decreased over time with significant differences shown in Figure 3a. Regarding the controls, the MRS media showed higher and statistically significant OD_600_ mean values compared to TSB (*p* < 0.0001, Figure 3a,b) considering *A. actinomycetemcomitans* and *S. mitis*. In *S. mutans*, instead, iMRS control was the only one showing a significantly higher value at all the tested times compared to TSB (*p* < 0.0001, Figure 3c), while iCysMRS displayed no significant differences with respect to TSB. After biofilm staining, the crystal violet (CV) retained was dissolved with 33% acetic acid and the absorbance was quantified at 570 nm. LBR01 CFS was completely ineffective in preventing *A. actinomycetemcomitans* biofilm formation, with significant differences versus all the other treatments and iMRS control (*p* < 0.0001 at all the time points, Figure 3d), while, when compared with iCysMRS and TSB controls, the only significant difference observed was the one at 48 h (*p* < 0.01; Figure 3d). LS03 CFS seemed to lose its effectiveness over time but without statistically significant differences. Among the controls, iCysMRS appeared to be the medium that better allowed biofilm formation, even though a significant difference was observed only with iMRS at all the time points (*p* < 0.0001, Figure 3d). Considering *S. mitis*, LBR01 CFS showed a similar activity to LS03 CFS, with the only significant difference between them at 48 h (*p* < 0.01; Figure 3e). LBR01 showed a significant difference against all the other CFSs at 48 and 72 h (*p* < 0.000 1 and *p* < 0.01, Figure 3e), while LS03 only at 72 h (*p* < 0.01 versus all, except *p* < 0.05 with *B. longum* 04 CFS, Figure 3e). All controls showed a significant difference when compared to CFS treatments at all endpoints (*p* < 0.0001, Figure 3e). Additionally, in this case, iCysMRS allowed a stronger biofilm formation, which was always significant against iMRS but not against TSB (*p* < 0.0001 and *p* < 0.05, respectively, only at 72 h, Figure 3e). In *S. mutans*, LBR01 CFS was the only treatment unable to prevent biofilm formation, showing significant differences with the other CFSs at all the time points (*p* < 0.0001, Figure 3f) but not against iCysMRS and TSB (ns; Figure 3f). All the controls were significantly different at all endpoints (*p* < 0.0001, Figure 3f), except for iCysMRS and TSB at 48 and 72 h (*p* < 0.05 and ns, Figure 3f). Figure 4 shows representative CV-stained biofilm images, which supports the biofilm results obtained through absorbance measurement (Figure 3d–f).

In conclusion, LBR01 CFS did not prevent *A. actinomycetemcomitans* and *S. mutans* biofilm formation, while, against *S. mitis*, it showed a similar activity to LS03 CFS, which was completely effective only against *S. mutans*, and it slightly lost its efficacy over time towards *A. actinomycetemcomitans*. All the other CFSs strongly reduced biofilm formation in all conditions. However, Wasfi and collaborators found that not only their *L. salivarius* ATCC 11741 CFS exhibited the highest antibiofilm activity against *S. mutans*, but also, together with *L. casei* ATCC 393, *L. reuteri* ATCC 23272, and *L. plantarum* ATCC 14917 CFSs, it reduced the expression of genes involved in exopolysaccharide production, acid tolerance, and quorum sensing [28]. Jaffar and colleagues demonstrated that both prokaryotic cells and CFSs of thirteen probiotic strains, including *L. casei* and *L. fermentum*, inhibited biofilm formation and induced biofilm degradation of three different *A. actinomycetemcomitans* strains [29]. Regarding the experiments with probiotic CFSs, the literature still lacks data related to their efficacy against *S. mitis*. On the other hand, works done on *A. actinomycetemcomitans* and *S. mutans* with the same probiotic species show conflicting results, underlining that the effects observed are strain specific. For this reason, after in vitro selection studies, such as this, it is important to better characterize probiotics and their CFS-specific action based on metabolomic analysis, to identify and isolate the single postbiotic molecules, which display an effect. 

### 2.4. Probiotic Effect on a Complex Bacterial Environment 

**Figure 5:**
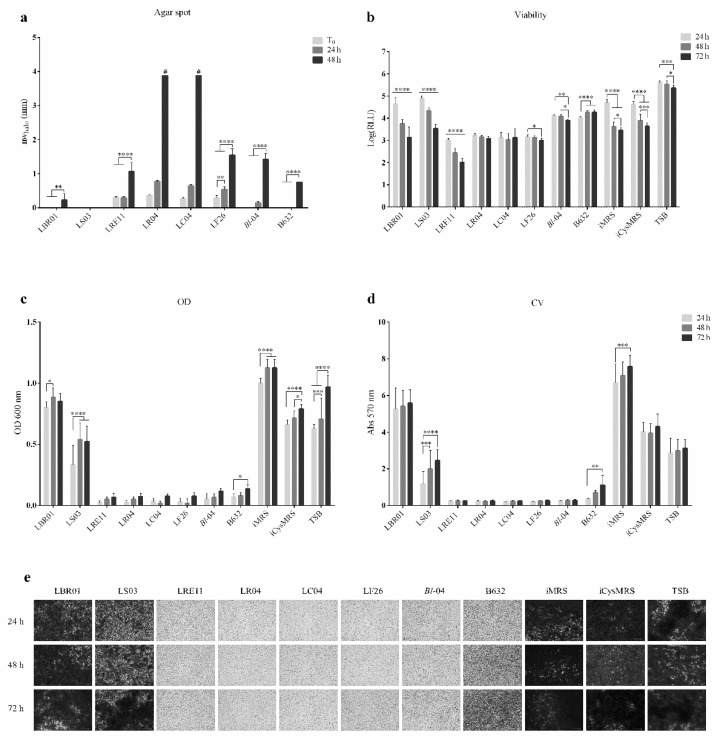
Live probiotic and CFS efficacy on a complex bacterial environment. (**a**) Normalized width measurements of inhibition halos obtained in the complex agar spot test. The diameters in mm of the inhibition halos were measured after 48 h of the three-pathogen incubation with the probiotic spots without (T_0_), or with 24 and 48 h pre-incubation. In the graph, the normalized width halo (nw_halo_) is expressed as the mean of three independent experiments ± SD. # complete growth inhibition zones. ** *p* < 0.01; **** *p* < 0.0001. (**b**) Viability assay. The pathogen-mix viability was determined after 24, 48, and 72 h of probiotic CFS treatment. Data are represented as the Log(mean) of three independent experiments ± SD. * *p* < 0.05; ** *p* < 0.01; *** *p* < 0.001; **** *p* < 0.0001. Log(RLU) = Logarithm10(relative luminescence unit); CFS = cell-free supernatant. (**c**) Optical density (OD) measurement at 600 nm and (**d**) Crystal violet (CV) biofilm quantification after CFS treatment of the complex pathogen environment. Data are represented as the mean of three independent experiments ± SD. * *p* < 0.05; ** *p* < 0.01; *** *p* < 0.001; **** *p* < 0.0001. OD 600 nm = optical density at 600 nm; Abs 570 nm = absorbance at 570 nm. (**e**) Representative CV-stained biofilm images obtained at FLoid^TM^ Cell Imaging Station with magnification 460×.

Both viable probiotic and their CFS effects were also investigated in a complex pathogen environment, obtained by mixing *A. actinomycetemcomitans*, *S. mitis*, and *S. mutans* in the same ratio. Results are shown in Figure 5. An additional agar spot test was also performed to observe whether the pathogens themselves could inhibit each other’s growth, with the result that no inhibition was observed (data not shown). The nw_halo_ measurements (mm) ± SD obtained in this assay are listed and represented in Table 2 and Figure 5a. LS03 never inhibited the growth of the three pathogens altogether, while LBR01 and B632 displayed this activity only when pre-incubated for 48 h. LRE11 showed a significant increase in its activity only at 48 h (*p* < 0.0001, Figure 5a), while LF26 displayed a mild increase at 24 h (*p* < 0.01, Figure 5a) and a higher one at 48 h (*p* < 0.0001, Figure 5a). LR04 and LC04 were the only two strains able to completely inhibit pathogen growth when pre-incubated for 48 h (*p* < 0.0001 at all the time points, Figure 5a). Against the pathogen co-cultures, the average of the inhibition zone diameters was lower when compared to the single strains, suggesting that pathogen–pathogen interactions can limit probiotic activity. To our knowledge, no agar spot data are available in the literature against bacterial co-cultures. 

Viability assay showed that LRE11 CFS was the most effective in inhibiting the three-pathogen co-culture viability, increasing its efficacy over time, showing significant differences with LBR01 and LS03 CFSs and the controls at 24 h (*p* < 0.0001, Figure 5b) and with all the treatments and controls at 48 and 72 h (*p* < 0.0001; Figure 5b). LC04, LR04, and LF26 CFSs showed a stable effect at all endpoints, with a decrease in the viability in LF26 CFS condition only at 72 h. No significant differences were recorded among them. LBR01 and LS03 CFSs did not show any reduction in the three-pathogen viability at 24 h, but their inhibitory effect on viability significantly improved over time. Anyway, LBR01 CFS was the one showing the lowest activity also in this co-culture condition, followed by LS03 CFS. *Bifidobacterium* CFS effects were generally worse than LAB CFSs at all endpoints (*p* < 0.0001, Figure 5b), except when compared with LBR01 and LS03 at 24 h. *B. longum* 04 CFS showed a significant difference against one of B632 only at 72 h (*p* < 0.0001, Figure 5b), while, at the other endpoints, they displayed a similar activity in reducing pathogen co-culture viability. The iMRS and iCysMRS controls showed similar viability levels with no significant differences between them at all measuring times. Both the above controls showed a significantly lower viability compared to the one of TSB at all endpoints (*p* < 0.0001, Figure 5b). 

When evaluating the three-pathogen biofilm formation, both OD_600_ and CV staining revealed that LBR01 and LS03 CFSs allowed it, with significant differences with all the other CFSs at all the tested times (*p* < 0.0001; Figure 5c,d). B632 CFS, in this complex assay, lost its preventive effect over time, showing a significant increase compared to LRE11, LR04, LC04, LF26, and *B. longum* 04 CFSs (*p* < 0.01, Figure 5d). Figure 5e shows representative pictures of the three-pathogen biofilm stained with CV. 

### 2.5. Co-Aggregation Assay

**Figure 6:**
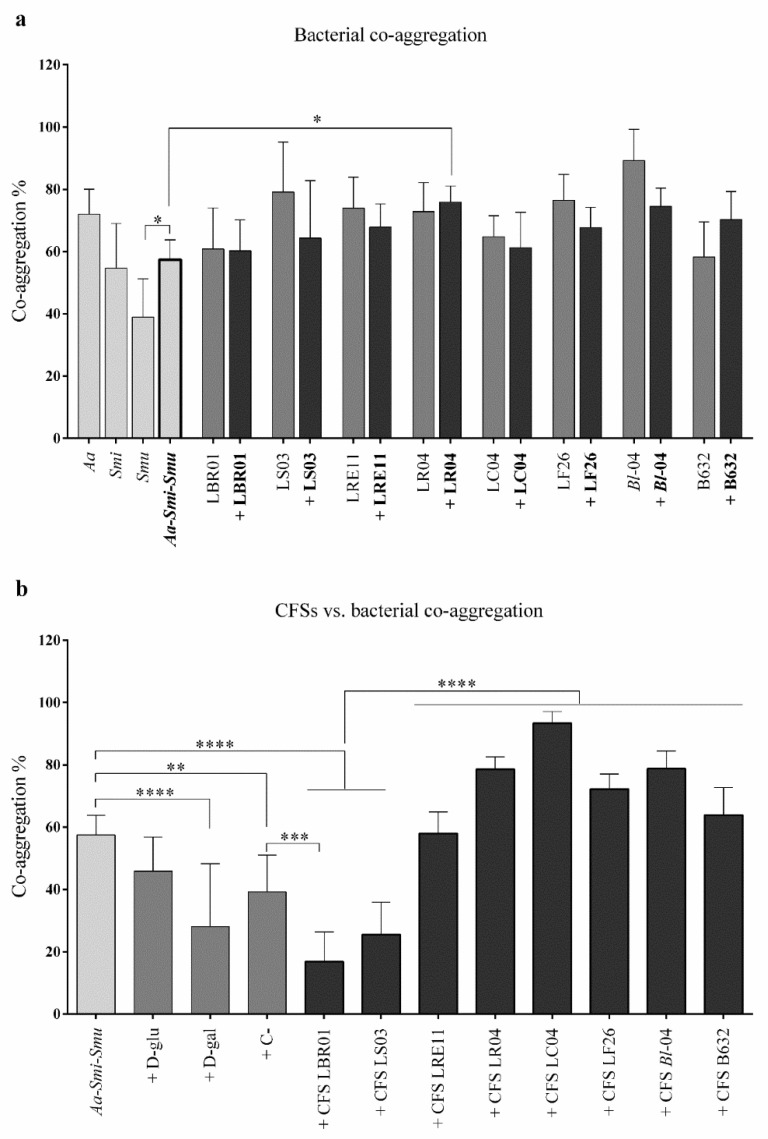
Co-aggregation assay. (**a**) Auto- and co-aggregation assay executed with pathogens and probiotics. The probiotic strain code alone refers to auto-aggregation; if a “+” before the probiotic strain is present, it means that the probiotic was added to the three-pathogen mix. (**b**) CFS effects on the three-pathogen co-aggregation. All data are represented as the mean of three independent experiments ± SD. * *p* < 0.05; ** *p* < 0.01; *** *p* < 0.001; **** *p* < 0.0001. Aa = *A. actinomycetemcomitans*; Smi = *S. mitis*; Smu = *S. mutans*; D-glu = D-glucose; D-gal = D-galactose; C- = negative control; CFS = cell-free supernatant.

The auto- and co-aggregation assays were developed to investigate single pathogen and probiotic strain interactions with themselves or with each other, and how probiotic CFSs could interfere with the three-pathogen co-aggregation. Figure 6a shows the auto-aggregation results obtained for each single bacterial strain, and the co-aggregation obtained after mixing the three pathogens with a probiotic strain. Among the individual pathogens, *S. mutans* showed the lowest auto-aggregation rate and was the only one exhibiting a significant difference with the three-pathogen co-aggregation values (*p* < 0.05, Figure 6a). Probiotic strains alone also showed an auto-aggregation ability and, when mixed with the pathogens, none of the strains displayed a significant effect when compared to the co-aggregation of the pathogens alone, except in the conditions + LR04, where the co-aggregation was significantly increased with respect to the three pathogens without the probiotic (*p* < 0.05, Figure 6a). Figure 6b shows the effect of CFSs on the three-pathogen co-aggregation ability. Among the controls, only D-galactose significantly reduced the pathogen co-aggregation rate (*p* < 0.0001, Figure 6b), showing also significant differences with the probiotic CFSs (*p* < 0.0001, Figure 6b) except the ones of LBR01 and LS03. The only CFSs showing a significant reduction in pathogen co-aggregation were those of LBR01 and LS03 (*p* < 0.0001, Figure 6b), with significant differences also with all the other CFSs (*p* < 0.0001), but the only one showing a significant difference with the C- was LBR01 (*p* < 0.001, Figure 6b). All the other CFSs showed a significant increase in pathogen co-aggregation (*p* < 0.001 for LR04 and *B. longum* 04; *p* < 0.0001 for LC04, Figure 6b), except the ones of LRE11 and LF26 that displayed no significant differences.

Interestingly, in the co-aggregation assay, only LBR01 and LS03 CFSs were able to inhibit the three-pathogen co-aggregation in a significant way, with LBR01 CFS showing a specific inhibition. The negative control used in this test was needed to check for nonspecific bacterial interactions, such as the ones due to hydrophobicity and ionicity, thus we could consider the inhibition specific when promoted by substances showing a significantly lower co-aggregation percentage when compared to the negative control [30]. An explanation as to why these two probiotic CFSs were not effective in the viability and biofilm formation assays, allowing bacterial growth and their interactions to form a biofilm, while they were in the co-aggregation assay, can be found in the experimental design and the CFS metabolite composition. While in the viability and biofilm formation assays pathogenic bacteria were plated in TSB, treated with CFSs, and incubated in optimal conditions for their growth, metabolizing, and adapting to the substances present in the CFSs, for this assay they were resuspended in CAB, that did not allow bacterial growth and adaptation, but it only facilitated molecules interactions. In addition, CFSs are complex substances, containing postbiotic molecules released by probiotics during their growth, and still some components of the MRS medium not digested by the bacteria that, altogether, can interfere with each other. This explanation can also be adapted to the other CFSs for which no effect in reducing the three-pathogens co-aggregation was observed, conversely leading to its significant increase, such as for LR04 and LF26 CFSs. This highlights again the importance of deeply investigating CFS composition, to identify the nature of the molecules responsible for a certain effect and isolate them from possible inhibitors.

On the other hand, when viable probiotics were used, no significant differences with the pathobiont co-aggregation were observed, except for a significant co-aggregation increase when LR04 was added to the pathogens. It can be assumed that since pathogens interact with probiotic cells, these ones may compete for cellular receptor binding, preventing pathogen cell adhesion, as also observed by Scillato and colleagues [31].

Our assumption is that assays such as the agar spot test and the viability and biofilm formation assays, are the most informative when the goal is to select probiotic strains for pathogen containment since they favour live bacterial interactions and pathogen adaptation to the substances, such as the antimicrobial compounds hydrogen peroxide and bacteriocins, organic acids, and enzymes present into the CFSs. In particular, developing these assays in a pathogen co-culture environment allows for re-creating an in vitro setting closer to the in vivo one, even though it has some limitations. In fact, these assays do not permit determining the specific pathogen interaction mechanisms and some constituents present in the oral microenvironment are not considered [32]. On the other hand, co-aggregation assay may be useful to question whether it is worth to deeper investigate some probiotic strains with a lower or no efficacy in the previous assays since it is a fast and cheap method.

## 3. Materials and Methods

### 3.1. Bacterial Cultures

*Aggregatibacter actinomycetemcomitans* (DSM 11123, Deutsche Sammlung von Mikroorganismen und Zellkulturen, DSMZ, Braunschweig, Germany), *Streptococcus mitis* (DSM 12643), and *Streptococcus mutans* (DSM 6178) were aerobically cultivated overnight (ON) at 37 °C and 200 revolutions per minute (rpm) in tryptic soy broth (TSB, Sigma-Aldrich, St. Louis, MO, USA, distributed by Merck Life Science S.r.l., Milan, Italy). The probiotic strains *Levilactobacillus brevis* LBR01 (DSM 23034), *Ligilactobacillus salivarius* LS03 (DSM 22776), *Limosilactobacillus reuteri* LRE11 (DSM 33827), *Lacticaseibacillus rhamnosus* LR04 (DSM 16605), *Lacticaseibacillus casei* LC04 (DSM 33400), *Limosilactobacillus fermentum* LF26 (DSM 33402) were aerobically grown in static conditions ON at 37 °C, using De Man, Rogosa and Sharpe broth (MRS, Condalab, distributed by Cabru S.A.S., Biassono, Italy), while the probiotic strains *Bifidobacterium longum* 04 (*Bl*-04 in figures and tables; DSM 24706) and *Bifidobacterium breve* B632 (DSM 23233) were cultured ON at 37 °C in anaerobic 2.5 L rectangular jars with Oxoid^TM^ AnaeroGen^TM^ sachets (Thermo Fisher Diagnostic S.p.A., Rodano, Milan, Italy) and using MRS supplemented with 0.5% N-acetyl-L-cysteine (Sigma-Aldrich). All the probiotic strains were kindly provided by Probiotical Research S.r.l., Novara, Italy. All the bacterial strains were freshly renewed before each experiment.

### 3.2. Agar Spot Test

The agar spot test was conducted to evaluate live probiotic strain efficacy in reducing oral pathogen growth. The protocol by Tejero-Sariñena et al. in 2012 was followed with a few modifications [33]. Three 10 µL drops of each probiotic strain were spotted onto a 1.5% agarized MRS medium plate and let dry at room temperature (RT). A semisolid pathogen suspension, obtained by diluting a fresh ON culture 1:1000 in TSB with 0.8% agar, was poured onto the spots immediately (T_0_), or after 24 and 48 h of spot incubation in proper conditions for probiotic growth. Then, the pathogen suspension was let solidify at RT before incubation at 37 °C for 48 h, when the inhibition halo diameters were measured. The same procedure was used to develop a more complex condition, testing the ability of live probiotics to inhibit the growth of the three pathogens altogether. Normalized width halo (nw_halo_) was calculated as shown by Marti M et al. [34]:

$${\mathrm{nw}}_{\mathrm{halo}}=\frac{d_{\mathrm{iz}}\;-\;d}{2\;\cdot \;d}$$

with d_iz_ = diameter of the inhibition zone (in mm) and d = spot diameter (in mm). In addition, the agar spot test was performed testing each pathogen against the others to determine whether they could inhibit their own growth. All experiments were independently repeated three times.

### 3.3. Probiotic Cell-Free Supernatant Production

To determine their postbiotic effect on pathogen viability and biofilm formation, probiotic cell-free supernatants (CFSs) were produced as reported by Squarzanti et al. in 2022 with few modifications [35]. Fresh strain cultures were inoculated with an optical density at 600 nm (OD_600_) = 0.05 into MRS and grown ON in proper conditions. Bacterial growth was assessed via OD_600_ measurement, then the cultures were centrifuged at 3000× *g* for 20 min at 4 °C (Heraeus Megafuge 16R, Thermo Fisher Scientific, Rodano, Milan, Italy). CFSs were then collected, sterilized with 0.22 µm PES filters (Clearline, distributed by Biosigma, Cona, Venice, Italy), aliquoted, and stored at −20 °C. MRS and MRS with cysteine were incubated as described for the probiotic cultures and used as controls in the following experiments (iMRS and iCysMRS, respectively).

### 3.4. Viability Assay

CFS-treated pathogen viability was assessed with BacTiter-Glo^TM^ Microbial Cell Viability Assay (Promega Italia S.r.l., Milan, Italy). Pathogens were seeded at OD_600_ = 0.01 (approximately 5 × 10^6^ CFU/mL) into a 96-well-plate, immediately treated with probiotic CFSs (50% *v*/*v*) and then incubated at 37 °C in static conditions. A plate for each pathogen and each time point of 24, 48, and 72 h was used. The viability assay was then performed following the manufacturer’s instructions and the luminescence was detected with a Spark microplate reader (Tecan Trading AG, Switzerland). A complex viability assay with pathogen co-culture was also optimized. Pathogens were plated altogether at the same OD_600_ = 0.01 and allowed to adapt for 1 h at 37 °C before CFS treatment. Then, the assay was executed as described above. In all the experiments, TSB, iMRS, and iCysMRS were used as controls. Each experiment was done with five replicates and repeated three times independently.

### 3.5. Biofilm Formation Assay

Pathogen biofilm formation level after CFS treatment was determined as previously published by Squarzanti et al. 2022, with few modifications [35]. Pathogens were plated independently at OD_600_ = 0.01 into a 48-well-plate and immediately treated with probiotic CFSs (50% *v*/*v*). A plate for each pathogen and each endpoint of 24, 48, and 72 h was used. OD_600_ was read before incubation (T_0_), and at each endpoint. After the OD_600_ reading, the biofilm was fixed with 4% paraformaldehyde (Bio-Optica S.p.A., Milan, Italy) for 30 min at RT. The supernatant was then removed, and the biofilm was stained with 1% crystal violet (CV) solution (Sigma-Aldrich) for 15 min at RT. Excess CV was removed by gently rinsing with tap water. Images were acquired with EVOS FLoid^TM^ Cell Imaging Station (Thermo Fisher Scientific, Waltham, MA, USA). To quantify the biofilm amount, CV was dissolved using a 33% acetic acid solution and its absorbance was read at 570 nm with a Spark microplate reader. A complex biofilm formation assay was also developed by plating pathogens altogether at the same OD_600_ = 0.01 and allowing them to adapt for 1 h at 37 °C before CFS treatment. The assay was then performed as described above. In all the experiments, TSB, iMRS, and iCysMRS were used as controls. Each experiment was done with four replicates and repeated three times independently.

### 3.6. Co-Aggregation Assay

Bacterial interactions were studied by developing auto- and co-aggregation assays, based on already published papers [36,37] with few modifications. Freshly renewed ON pathogen and probiotic cultures were centrifuged at 3000× *g* for 15 min at RT (Heraeus Megafuge 16R), then, the pellet of each culture was resuspended at OD_600_ = 1 in co-aggregation buffer (CAB; 150 mM NaCl, 1 mM Tris, 0.1 mM CaCl2, 0.1 mM MgCl2 2H2O). Auto-aggregation was determined using 1 mL aliquots of bacterial suspension, while for co-aggregation assay equal amounts of pathogens, or pathogens and probiotics, were mixed into a tube and vortexed 30 s before aliquoting. The OD_600_ of each aliquot was read immediately (T_0_) and after 8 h (T_8_) incubation at RT (NanoPhotometer NP80; Implen, Munich, Germany). CFSs were also used to assess their activity in inhibiting pathogen interactions (50% *v*/*v* with the pathogen mixture). The following conditions were used as controls: auto-aggregation values of both pathogens and probiotics; D-glucose (D-glu) and D-galactose (D-gal) solution in CAB (50 mM final volume) as positive controls; tween-20 0.05% in 0.2 M NaCl as negative control (C-), since it inhibits non-specific bacterial interactions [30]. The following equation was used to calculate the aggregation percentages:

$$\mathrm{auto}-\;\mathrm{or}\;\mathrm{co}-\mathrm{aggregation}\;\%=\frac{{\mathrm{OD}}_{600}{\;T}_{0\;}{-\;\mathrm{OD}}_{600}T_{8}}{{\mathrm{OD}}_{600}{\;T}_{0}}\;\cdot 100$$

with OD_600_ T_0_ = OD at T_0_ and OD_600_ T_8_ = OD at T_8_. Each experiment was done with three replicates and independently repeated three times.

### 3.7. Statistical Analysis

One-way and two-way ANOVA tests, with Tukey post-hoc correction, were performed using GraphPad Prism version 6.01 for Windows (GraphPad Software, San Diego, CA, USA, www.graphpad.com (accessed on 15 June 2018)). Results were represented as the mean of the replicates ± standard deviation (SD). Significant differences were considered for *p* < 0.05.

## 4. Conclusions

Assessing probiotic effect against pathogens, by comparing both the viable strains and their metabolic products, and using pathogen co-cultures, permits understanding whether a probiotic can be used in case of infection or, when not possible, if its postbiotics can be employed still guaranteeing the same efficacy. As seen, bacterial behaviour is influenced by the mutual relationship among micro-organisms and by in vitro culture conditions, as it also happens in vivo, and conditioned by the environmental and antibiotic pressures, which can select them towards commensalism or pathogenicity and accelerate the onset and course of local and systemic diseases. By means of this screening, important information on the efficacy of viable probiotic strains and that of their CFSs was collected, selecting LRE11, LR04, LC04, and LF26 as the most efficient in oral pathogen containment. Nevertheless, deeper investigations are needed to better elucidate their in vivo interactions and employability in human patients.

## Figures and Tables

**Table 1:** Normalized width measurement in mm of inhibition halos obtained in the agar spot test.

		Probiotic Spot Incubation Time (h)		
Pathogen	Probiotic Strain	0	24	48
*A. actinomycetemcomitans*	LBR01	-	1.19 ± 0.17	2.17 ± 0.4
	LS03	-	-	-
	LRE11	0.23 ± 0.04	0.23 ± 0.04	0.96 ± 0.16
	LR04	0.58 ± 0.07	1.38 ± 0.13	>3.88 ^#^
	LC04	0.46 ± 0.10	1.17 ± 0.31	2.46 ± 0.14
	LF26	0.48 ± 0.07	1.19 ± 0.22	1.98 ± 0.34
	*Bl*-04	-	1.65 ± 0.13	>3.88 ^#^
	B632	-	1.13 ± 0.11	2.27 ± 0.32
*S. mitis*	LBR01	-	0.31 ± 0.00	0.83 ± 0.26
	LS03	-	-	-
	LRE11	0.27 ± 0.04	0.21 ± 0.04	0.44 ± 0.06
	LR04	0.50 ± 0.06	0.90 ± 0.18	1.38 ± 0.13
	LC04	0.33 ± 0.04	0.77 ± 0.04	1.17 ± 0.07
	LF26	0.27 ± 0.04	0.58 ± 0.16	0.92 ± 0.19
	*Bl*-04	-	0.88 ± 0.11	1.60 ± 0.18
	B632	-	0.46 ± 0.10	1.17 ± 0.10
*S. mutans*	LBR01	-	0.83 ± 0.07	>3.88 ^#^
	LS03	-	-	-
	LRE11	0.35 ± 0.07	0.48 ± 0.04	0.98 ± 0.16
	LR04	0.71 ± 0.07	1.52 ± 0.25	>3.88 ^#^
	LC04	0.77 ± 0.16	1.92 ± 0.34	>3.88 ^#^
	LF26	0.52 ± 0.04	0.88 ± 0.11	>3.88 ^#^
	*Bl*-04	-	1.67 ± 0.29	>3.88 ^#^

Data are reported as the mean of three independent measurements ± SD.—no inhibition halo observed; # complete growth inhibition was observed.

**Table 2:** Normalized width measurement in mm of inhibition halos obtained in the complex agar spot test.

	Probiotic Spot Incubation Time (h)		
Probiotic Strain	0	24	48
LBR01	-	-	0.23 ± 0.18
LS03	-	-	-
LRE11	0.29 ± 0.04	0.29 ± 0.04	1.06 ± 0.27
LR04	0.35 ± 0.04	0.77 ± 0.04	>3.88 ^#^
LC04	0.27 ± 0.04	0.65 ± 0.04	>3.88 ^#^
LF26	0.29 ± 0.07	0.54 ± 0.07	1.54 ± 0.19
*Bl*-04	-	0.15 ± 0.04	1.42 ± 0.18
B632	-	-	0.75 ± 0.00

Data are reported as the mean of three independent measurements ± SD.—no inhibition halo observed; # complete growth inhibition was observed.

## Data Availability

All relevant data are within the manuscript.
